# Hook Plate Technique for Bony Mallet Thumb

**DOI:** 10.1155/2019/3538405

**Published:** 2019-11-04

**Authors:** Fumihiro Mukasa, Yoshimasa Tomita, Hideyuki Hirasawa, Kazuo Kaneko

**Affiliations:** ^1^Department of Orthopedic Surgery, Tokyo Rosai Hospital, 4-13-21, Omori-minami, Ota-ku, Tokyo 143-0013, Japan; ^2^Department of Orthopedic Surgery, Juntendo University School of Medicine, 3-1-3, Hongo, Bunkyo-ku, Tokyo 113-8431, Japan

## Abstract

Bony mallet is a common sport injury, but bony mallet thumb is rarely encountered. We performed open reduction and fixation of bony mallet thumb using a hook plate procedure on a 27-year-old man under general anesthesia. The patient began working one day after surgery. Six months postoperatively, the patient had excellent dexterity according to Crawford's evaluation criteria and no difficulties at work or playing softball. Tension band fixation, compression pins, and the extension block technique are commonly used to manage bony mallet. Despite the anatomical reduction, rigid fixation, and early resumption of motion skills offered by the hook plate technique, to our knowledge, no previous reports of its application to bony mallet thumb were found. In this case, the hook plate technique was chosen and made an early return to work possible and brought about a successful result.

## 1. Introduction

Mallet finger is a common sport injury, but bony mallet thumb is rarely encountered because the thumb is shorter than the other fingers and the extensor pollicis longus (EPL) is thicker than the other extensors [1]. It is reported that bony mallet thumb has been managed by tension band fixation, compression fixation pins, or the extension block technique [2–4]. The anatomical reduction, rigid fixation, early resumption of motion activity, and early return to the work offered by the hook plate technique for bony mallet finger have not previously been reported in bony mallet thumb injuries, so far as we know. This is the first report that the hook plate technique was applied to the bony mallet thumb.

## 2. Case Report

**Figure 1:**
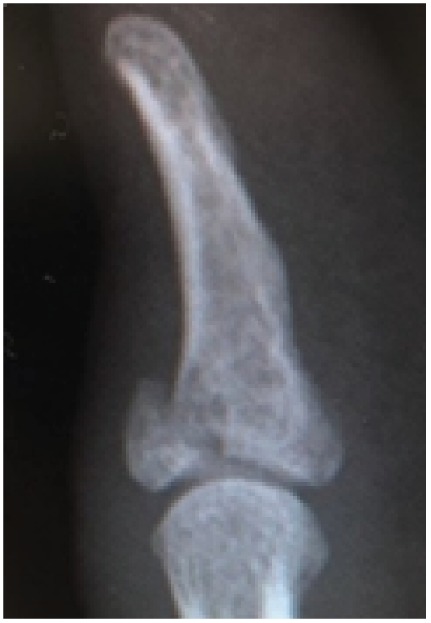
Lateral radiograph at the injury site.

**Figure 2:**
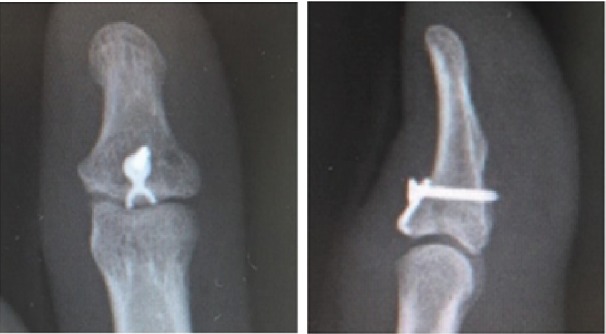
The radiograph images after reduction and fixation.

**Figure 3:**
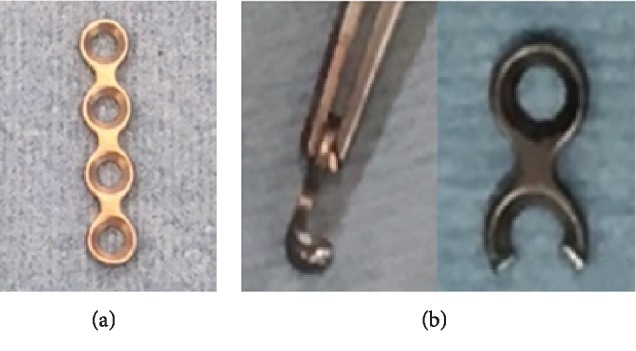
The 1.5 mm mini plate before (a) and after the plate was modified by cutting the hole and bending the ends of crescent arc (b).

The patient was a 27-year-old man who sprained his right thumb while playing softball, resulting in tenderness and swelling around the interphalangeal (IP) joint. He was a software engineer, and his dominant hand was the right hand. Physical examination revealed complete loss of active extension and also that passive extension and active flexion of the IP joint were full. The IP joint was stable against radial and ulnar stress. The images of plain radiography and computed tomography showed an avulsion fracture of one-third to half of its articular surface at the base of the distal phalanx (Figure 1). Using the hook plate technique, open reduction was performed under general anesthesia 19 days after the injury (Figure 2). An incision was made along the crease on the dorsal side of the IP joint. The fracture was reduced and fixed using a hook plate adapted from a 1.5 mm micro module (KLS Martin, Germany, Figure 3(a)). One of the holes was cut, and one-third of its circumference was removed. The ends of the crescent arc were bent approximately 100° by pliers to form two sharp pointed hooks (Figure 3(b)). Two small longitudinal incisions were made at the EPL. The hook plate was slid under the nail matrix, the hooks were inserted through the slips, and the bone fragment was gripped. The screw was inserted into the plate hole to add compression to the fragment. The articular congruency and stability of the fragment in the direction of passive flexion were checked, and the wound was closed.

**Figure 4:**
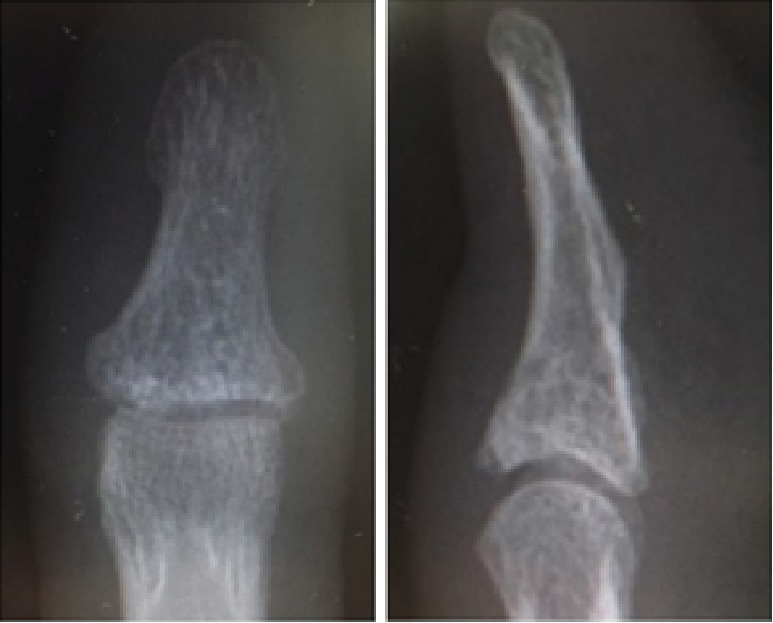
Follow-up radiographs showing the healed fracture.

**Figure 5:**
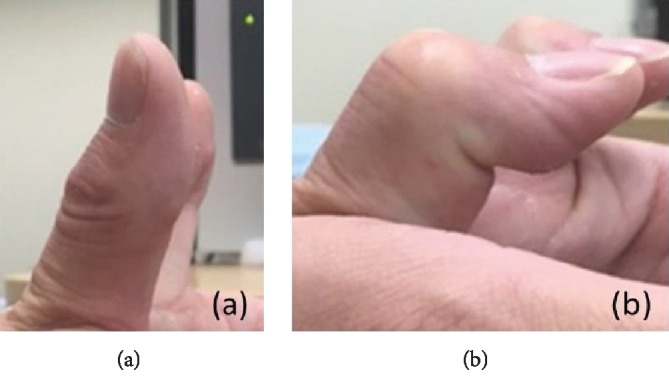
Active range of motion of the IP joint of 8° of extension (a) and 90° of flexion (b).

**Figure 6:**
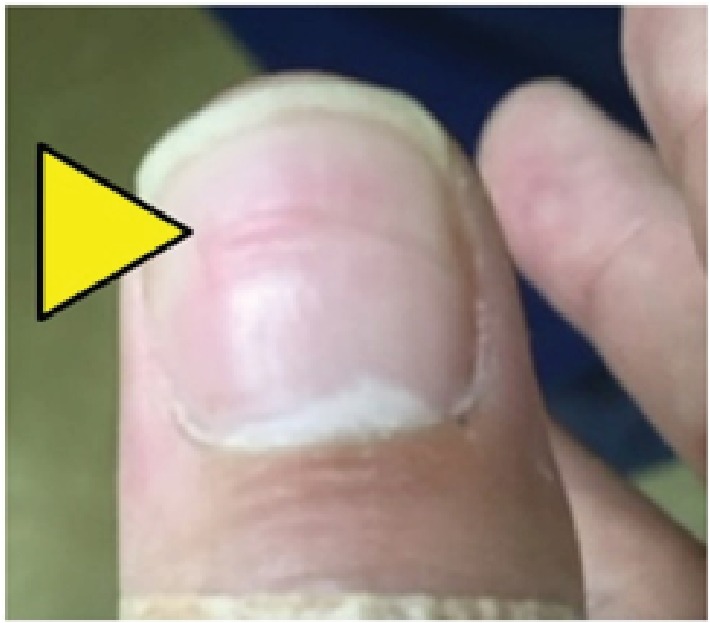
Transverse line deformity of the nail that gradually disappeared.

The splint was maintained for 1 week before motion of the IP joint was allowed, and the patient returned to work 1 day after surgery. Bone union was achieved and the plate was removed 3 months after the procedure (Figure 4). At the last follow-up of 6 months after surgery, the active range of motion at the IP joint was 80 degrees of flexion and 8 degrees of extension. The range of motion on the contralateral side was 85 degrees of flexion and 10 degrees of extension (Figures 5(a) and 5(b)). The patient had a transverse line nail deformity (Figure 6) that gradually improved. According to Crawford's evaluation criteria [5], the result was excellent. The patient experienced no injury-related difficulties both at work and while playing softball.

## 3. Discussion

Bony mallet thumb is a very rare injury, only one in 160 mallet fractures according to Wehbe and Schneider [6]. The thumb is protected because it is shorter than the other fingers and the EPL is thicker than those of other fingers [1]. Bony mallet thumb requires more precise reduction and more rigid fixation than the other bony mallet fingers because the thumb's terminal tendon is short, approximately 4 mm [7], and the range of extension at the IP joint is larger than those of the other DIP joints. Therefore, extension lag can easily result from deformity of the IP joint surface.

The surgical options for bony mallet thumb include tension band fixation, compression fixation pins, and the extension block technique [2–4]. Teoh and Lee and Theivendran et al. have described the use of the hook plate technique for bony mallet finger, but not bony mallet thumb, to obtain anatomical reduction and stable fixation with early mobilization [8, 9]. In this patient, the hook plate technique was used to repair bony mallet thumb and achieve satisfactory anatomical reduction and rigid fixation without requiring percutaneous pins, which allowed early resumption of motion activity. He was a software engineer and was able to resume work which required the use of a computer keyboard. The extension block technique (Ishiguro's method) and its modifications are very good options for the bony mallet finger, because they were reported to be easier than open reduction and obtain successful results [10]. But the extension block technique cannot allow early mobilization and requires meticulous percutaneous pin care. So, if the extension block technique had been used, he would not have resumed work so early. When considering early resumption of work, the hook plate technique seems to be a good indication.

**Figure 7:**
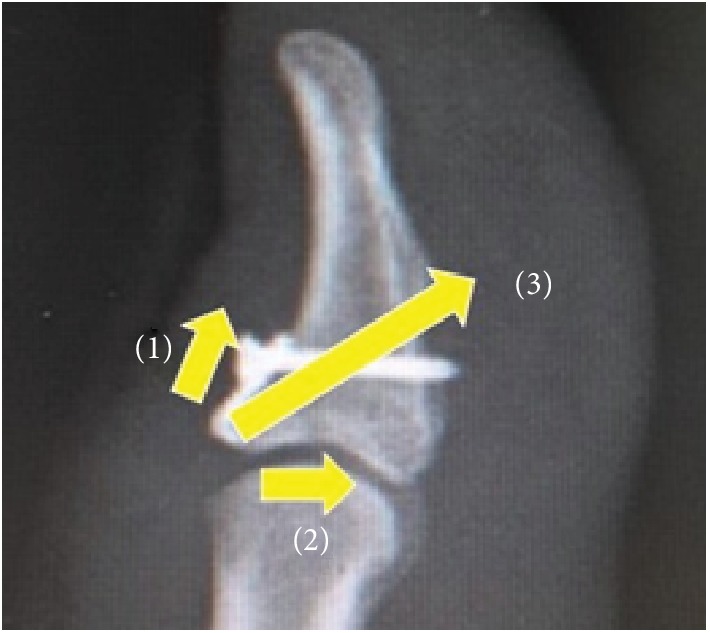
The mechanism of compression force generation involved. (1) Force in the distal direction produced by the hook. (2) Force in the horizontal direction produced by the screw and plate. (3) Compression force is applied to the fragment.

The hook plate technique has advantages that include control and anatomical reduction of even large dorsal fragments and can be used to manage thin or avulsion fractures. It can also apply compression and stability to the fragment. The hook produces the force in the distal direction (Figure 7 (1)), the screw and the plate produce the force in the horizontal direction (Figure 7 (2)), and they are combined to be a compression force applied to the fragment (Figure 7 (3)). The hook plate technique permits early resumption of joint motion and does not require meticulous percutaneous pin care [8]. The reported postoperative complications of the hook plate technique include articular cartilage damage, nail deformities, extensor tendon lesions, and skin irritation [8]. Damage of the articular cartilage of the middle phalanx head may result from inadequate length or shape of the hook. In this patient, the hook was placed into the distal fragment through the EPL and articular cartilage damage did not occur. Nail deformities can occur following damage of the nail matrix by the screw or the plate, but the deformity that occurred in this patient did not occur when the plate was located. The deformity may have happened at the time of injury. The nail matrix is located close to the bony insertion of the terminal extensor tendon. The distance from the terminal extensor tendon insertion to the proximal edge of the germinal nail matrix is approximately 1.2 mm [11]. If the hook plate rests on the nail bed, damage of the germinal nail matrix will produce nail deformity. As the hook plate was placed under the nail matrix, the chance of nail deformity was minimized.

Teoh and Lee reported stable fixation and early mobilization with the use of the hook plate technique in mallet finger [8]. However, to the best of our knowledge, no previous cases have reported the use of the hook plate technique for bony mallet thumb. According to this case experience, the hook plate technique can be recommended as an option for the treatment of bony mallet thumb. Further follow-up is needed to assess the long-term benefits of this technique for managing mallet thumb, including the incidence of osteoarthritis. In conclusion, although not a minimally invasive surgery, the hook plate technique offers good anatomical reduction and rigid fixation. It allows early resumption of motion and provides satisfactory results in a patient with bony mallet thumb. And it is considered that when the early return to work is required, the hook plate technique seems to be a good indication.
